# Annus horribilis: pandemic, infodemic and our response

**DOI:** 10.4314/ahs.v20i4.1

**Published:** 2020-12

**Authors:** James K Tumwine

**Affiliations:** Editor in Chief, African Health Sciences

This December 2020, bumper harvest, issue of *African Health Sciences* is unique in many ways: it ends the year that might be described using the Queen of England's own words: *annus horribilis*.^1^

It has been a horrible year indeed with record numbers of lives lost to Covid-19; record numbers of papers published on one disease in less than a year: a whooping 750000 papers on Covid-19 alone and still counting. What is disturbing, though, is that most of these papers are not very far from *fake news* or rather very poor science: anecdote, case series, poorly designed studies etc. With so much genuine hysteria about such a horrible pandemic there has emerged, inevitably, what WHO calls an *infodemic*.^2^ While there is a concerted effort to stem the pandemic by the development of vaccines, there has not been such a strong effort to immunize our population against misinformation.^3^ The era of unlimited social media and the world wide web in general has not helped. Editors in mainstream health journals have a responsibility to stem this. Sieving the proliferating submission of manuscripts on Covid_19 is our responsibility. In the 6 months ending September 30^th^ alone, AHS has received over 2000 manuscripts double the number we would expect in a year! This has placed enormous pressure on all our volunteer establishment and has inevitably meant increased rejection of manuscripts to the annoyance of our authors. The process of peer review e.t.c, has also slowed down with the expected consternation.

In this issue we purposely have only two papers on Covid_19.

In the first one, Emirate scientists^4^ have written an opinion piece on vaccines for Covid-19. On the other hand, Dr. Oliver Ombeva Malande,^5^ a Kenyan paediatrician working in Uganda, recounts his personal experience with Covid-19 that he contracted from a child he was treating, busting the myth about Covid-19 and child health.

We do apologize to our stakeholders for the process slow down. We hope that you will enjoy this bumper issue none the less.
